# Acute liver toxicity with ifosfamide in the treatment of sarcoma: a case report

**DOI:** 10.1186/1752-1947-5-180

**Published:** 2011-05-13

**Authors:** Michelle CM Cheung, Robin L Jones, Ian Judson

**Affiliations:** 1 Liver Unit, King's College Hospital, Denmark Hill, London SE5 9RS, UK; 2 Medical Oncology Division, University of Washington, Fred Hutchinson Cancer Research Center, Seattle, WA, USA; 3 Sarcoma Unit, Royal Marsden Hospital, Fulham Road, London SW3 6JJ, UK

## Abstract

**Introduction:**

Ifosfamide is a chemotherapy agent infrequently associated with liver toxicity. To the best of our knowledge, this report is the first to describe serious liver toxicity associated with ifosfamide used in combination with doxorubicin that caused acute but fully reversible liver failure and encephalopathy. This report reviews the possible mechanisms by which ifosfamide causes this adverse effect.

**Case report:**

A 61-year-old Caucasian woman who presented with an inoperable right neck mass due to synovial sarcoma was treated with standard-dose ifosfamide and doxorubicin. Within 24 hours of completing the first cycle of chemotherapy, she developed significant derangements in liver function, with a 250-fold increase in transaminase and associated synthetic function impairment and encephalopathy. No other causes of liver failure were identified. Both biochemical tests and encephalopathy were reversed after supportive management and treatment with *N*-acetylcysteine. No liver toxicity was observed with subsequent cycles of chemotherapy with doxorubicin alone.

**Conclusion:**

This case highlights the possibility that chemotherapy agents can cause rare and idiosyncratic toxicities, so physicians must be vigilant for drug reactions, especially when patients do not respond to usual treatment.

## Introduction

Ifosfamide is an alkylating cytotoxic agent used in the treatment of a variety of cancers, including germ cell tumors, sarcomas, lymphoma and lung cancer. It is used alone or, more frequently, in combination with other drugs, such as in the present case, with doxorubicin. The dose-limiting toxicities of ifosfamide are myelosuppression and urotoxicity. Effects on the liver are infrequently seen, and significant toxicity has been suggested in only one case report so far [1]. Manufacturers suggest a 3% incidence of hepatic function derangement with the use of ifosfamide as a single agent on the basis of 30 single-agent studies of 2070 patients in the published literature [2]. Table 1 shows a list of adverse drug reactions listed on the product package insert. This case report describes the use of ifosfamide in a patient with synovial sarcoma, a common type of soft tissue sarcoma with notable sensitivity to chemotherapy [3].

**Table 1:** Significant adverse effects of ifosfamide^a^

Adverse reaction	Incidence, %
Alopecia	83%
Nausea/vomiting	58%
Hematuria	46%
Gross hematuria	12%
Central nervous system toxicity	12%
Infection	8%
Renal impairment	6%
Liver dysfunction	3%
Phlebitis	2%
Fever	1%

^a^Adverse effects are as listed on the product package insert.

## Case presentation

A 61-year-old Caucasian woman with a diagnosis of synovial sarcoma in the right lung apex initially presented with a six-month history of increasing scapular pain, for which she was undergoing physiotherapy. During this time, a neck mass developed which progressed to vocal hoarseness, right-sided Horner's syndrome and arm swelling. She was an otherwise well woman with no significant medical history or drug history.

She underwent a computed tomography (CT)-guided biopsy, which led to the diagnosis of synovial sarcoma. Staging investigations confirmed a 10 cm × 8 cm × 9 cm mass extending from the carina to the right side of the neck and causing tracheal deviation and compression of the internal jugular vein. There was no metastasis seen on the staging CT scan or on the positron emission tomography (PET) scan.

The localized but extensive tumor was clearly inoperable, and the treatment plan was to initiate chemotherapy and subsequently consolidate this treatment with radiotherapy or surgery. She was commenced on the standard chemotherapy combination of ifosfamide at 3 g/m^2^/day for three days and doxorubicin at 20 mg/m^2^/day for three days. Her body surface area was 1.6 m^2^, so she received a total of 4800 mg/day ifosfamide infused over four hours. Other medications co-administered with the chemotherapy regimen were dexamethasone, ondansetron and metoclopramide as anti-emetics and mesna for the prevention of urothelial toxicity.

Within 24 hours of completing the three-day treatment, the patient became drowsy and complained of hallucinations. This occurrence was thought to be a manifestation of ifosfamide neurotoxicity, a well-established side effect. Methylene blue at 50 mg three times daily was administered intravenously as the standard antidote.

Despite this treatment, her confusion persisted and blood tests revealed that at day one after finishing chemotherapy, she had developed a dramatic deterioration in her liver function tests, with a 250-fold rise in alanine aminotransferase (ALT) as well as abnormal liver synthetic function and renal function (Table 2). The development of encephalopathy within seven days of the onset of jaundice constitutes hyperacute liver failure. She had a modestly elevated ammonia level at 77 μM/l (normal, < 50) accompanying the encephalopathy. These findings were attributed to ifosfamide use on the basis of the temporal relationship and the subsequent normalization after drug withdrawal. Apart from her mental state, her clinical examination was unremarkable. In particular, there was no fever, rash or arthralgia to suggest a hypersensitivity drug reaction. The clinical features of drug-induced liver failure are difficult to differentiate from acute liver failure of other etiologies.

**Table 2:** Blood results and number of days post-completion of chemotherapy^a^

Number of days post-chemotherapy	ALT, IU/l (normal range, < 40)	AST, IU/l (normal range, 10 to 42)	ALP, IU/l (normal range, 24 to 110)	GGT, IU/l (normal range, < 35)	Br, μM/l (normal range, < 17)	INR (normal = 1)	Albumin, g/l (normal range, 30 to 50)	Creatinine, μM/l (normal range, 54 to 98)
Baseline	12		75	19	11		39	64
1	621		71		38	1.9	29	76
2	3086	5209	77	27	24	2.2	29	107
3	1894	1539	70	35	22	1.6	27	127
4	1203	383	72	52	21	1.3	26	122
5	792	123	76	83	27	1.2	27	112
6	630	68	85	102	23	1.1	27	101
7	443	39	88	103	25	1.1	26	87
8	309	30	86	95	19	1.1	28	84
9	229	25	94	88	18	1.0	30	78
10	148	20	82	85	13	1.0	29	89
11	102	16	84	78	12	1.0	27	93
12	81		100	77	8	1.0	29	86

^a^IU, international normalized ratio; ALT, alanine transferase; AST, aspartate transaminase; ALP, alkaline phosphatase; GGT, γ-glutamyl transpeptidase; Br, bilirubin.

To exclude other causes of acute liver failure, a full liver screen was carried out, all of which produced negative results (ceruloplasmin, auto-antibodies, ferritin, viral hepatitis screen, α-fetoprotein and paracetamol levels). An ultrasound of the liver showed normal venous flow with no focal lesions, fatty infiltration or underlying chronic liver pathology. A brain CT scan excluded intracerebral causes of acute confusion and showed a normal brain without significant cerebral edema, which may be associated with higher grades of hepatic encephalopathy.

All concurrent medications were stopped. These drugs included cocodamol 30/500 (a combination of codeine phosphate and acetaminophen), Oramorph (oral morphine sulfate) and amitriptyline to prevent any additional sedative effects. The patient also regularly took pantoprazole and multivitamins, which were withheld. Proton pump inhibitors have been reported rarely or very rarely to produce jaundice and hepatitis. However, the patient had not previously experienced any deleterious effects from taking these medications.

The patient was treated with *N*-acetylcysteine at 150 mg/kg over 16 hours, which commenced on the second day post-chemotherapy for a total of four days until her hepatic function and enzyme tests improved. Although *N*-acetylcysteine is the antidote for paracetamol overdose, there is evidence for its use in other forms of drug-induced acute liver failure to reduce mortality [4]. She was also given lactulose regularly at 20 ml twice daily to reduce encephalopathy. She remained hemodynamically stable throughout this period and did not require high-dependency care or mechanical ventilation.

She had biochemical improvements after commencing treatment (and stopping the offending chemotherapy agent). Her level of encephalopathy gradually receded to mild somnolence, and by about 10 days post-treatment she felt completely back to normal. Of note is that her transaminase levels had not yet normalized and her alkaline phosphatase level had not peaked at that point.

After recovery, the patient subsequently received chemotherapy with doxorubicin alone and experienced no further derangements in liver function. She received the same regimen of anti-emetics as for the first cycle (dexamethasone, ondansetron and metoclopramide), so although ondansetron has been associated with transient elevation of liver enzymes as a rare side effect, these medications were not thought to be to blame for the hepatotoxicity observed. Mesna, which was not required with the omission of ifosfamide, has no reported side effects on the liver recorded in the British National Formulary or in a survey of over 100 participants in controlled trials [2].

The patient demonstrated a good partial response after six cycles of doxorubicin treatment and then underwent consolidation radiotherapy to the right lung apex. She is being followed up every three months with alternating chest X-rays and chest CT and is symptomatically well.

## Discussion

Liver injury during chemotherapy may not always reflect direct hepatotoxicity of anti-cancer drugs. It may be due to or exacerbated by tumor disease and progression, immunosuppression, concurrent medical problems, nutritional deficits or parenteral feeding and polypharmacy, which can affect susceptibility to acute liver insult. Most hepatotoxic reactions associated with chemotherapy agents are idiosyncratic, due to immunological reactions or variations in host metabolic response, and not dose-dependent [5]. Immune-mediated drug reactions tend to show a latency of one to five weeks and are associated with hypersensitivity features such as fever, rash, eosinophilia and autoantibody positivity. Metabolism-mediated reactions lack these features. Biopsy of the liver in acute drug reactions may show cytolytic or cholestatic features or evidence of vascular injury [6]. While certain drugs are known to cause specific types of injury, the type of injury in idiosyncratic drug reactions can be of variable morphology. A liver biopsy can be performed when drug-induced liver injury is suspected, along with imaging and laboratory investigations to exclude other causes, but the key to the diagnosis is the temporal relationship of drug exposure and the patient's clinical picture. A validated diagnostic scale has been developed to aid in the diagnosis of drug-induced liver injury. It is based on the time correlation with drug use and withdrawal, response to re-exposure, previous reports of liver injury and exclusion of alternative causes [6].

While ifosfamide and its structurally related alkylating agent, cyclophosphamide, are both activated in the liver by P450 oxidases, they are uncommon hepatic toxins [7], and it is suggested they can be used safely in patients with abnormal liver function without the need to modify dosage [5]. One paper has suggested a dose reduction of 25% for bilirubin > 3 mg/dl [8]. A few reports of hepatotoxicity associated with the use of cyclophosphamide have been published [5]. This adverse reaction is thought to be due to the metabolites of cyclophosphamide, particularly acrolein, which, unusually, is a dose-dependent effect. Cyclophosphamide in high doses, such as those used in bone marrow pre-conditioning, can also lead to veno-occlusive disease [6]. While ifosfamide also produces acrolein as a metabolite, it has not been reported to be associated with hepatotoxicity. One report has described a patient with breast cancer and extensive liver metastases, but no pre-treatment deterioration in liver function, who developed acute liver failure and subsequently died after treatment with ifosfamide and docetaxel, although there was also a rise in uric acid and tumor lysis could have contributed to the patient's death [1]. The present case report is the first recorded instance of significant liver toxicity associated with ifosfamide use in combination with doxorubicin.

**Figure 1:**
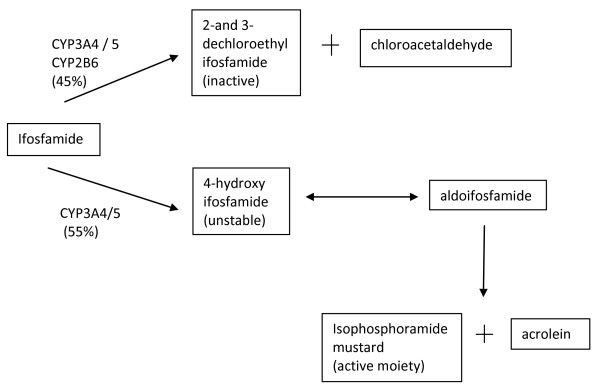
**Simplified diagram of ifosfamide metabolism**. Ifosfamide is a prodrug which is converted by P450 enzymes into the inactive decholoroethyl-ifosfamide and 4-hydroxy-ifosfamide, which exists in equilibrium with its tautomeric form aldoifosfamide. This spontaneously decomposes into active isophosphoramide mustard. The proportion of dechloroethylation required to produce chloroacetaldehyde is higher in ifosfamide (45%) than in cyclophosphamide (10%). Adapted from Zhang *et al*. [7] and Tascilar *et al*. [9].

Ifosfamide is associated with more commonly known side effects, which can be explained by its metabolism (Figure 1). It causes myelosuppression which is dose-dependent and ameliorated by the use of growth factors such as granulocyte colony-stimulating factor (G-CSF). G-CSF was not used in our case. Urotoxicity is manifested through hemorrhagic cystitis due to acrolein, which is prevented by vigorous hydration and co-administration of mesna, which reacts with acrolein. Nephrotoxicity as characterized by Fanconi syndrome and glomerular damage is more common in children and is much more prevalent in association with ifosfamide than with cyclophosphamide [7]. Approximately 45% of the therapeutic dose of ifosfamide is metabolized into chloroacetaldehyde (CAA) via *N*-dechloroethylation, whereas only 10% of cyclophosphamide is converted to CAA [9]. Thus, the nephrotoxicity of ifosfamide has been attributed to this metabolite. Chloroacetaldehyde is also thought to be responsible for ifosfamide-induced encephalopathy. Structurally, it is related to chloralhydrate, a known hypnotic. Methylene blue may counteract the oxidation reactions in the mitochondria that are linked to CAA and thought to be responsible for encephalopathy [10].

It is possible that ifosfamide use in susceptible individuals may cause hepatotoxicity via acrolein. However, if acrolein is implicated, then mesna should have a role in protecting against liver injury. Our patient did not have any evidence of urothelial toxicity, such as hematuria, to indicate a failure of mesna in neutralizing the acrolein produced. However, an idiosyncratic drug reaction to ifosfamide may also involve a completely different pathway due to individual metabolic variance. Measurement of ifosfamide metabolites requires specific liquid chromatography techniques and is not readily available outside research studies, but in this case the demonstration of an unusually high level of acrolein, for example, may have been of clinical value to elucidate the mechanism of idiosyncratic hepatotoxicity.

The main treatment of drug-induced liver toxicity is withdrawal of the offending agent. Most patients recover completely; therefore, management involves providing supportive care in the interim. Some patients may require plasmapheresis. Corticosteroids have no established role, but may be used in suppressing the hypersensitivity features of immunological idiosyncratic reactions [6]. *N*-acetylcysteine may help by replenishing liver glutathione stores [4]. Ultimately, acute liver failure may necessitate transplantation.

## Conclusion

Idiosyncratic drug reactions are rare and unpredictable. This case report describes the previously undocumented hepatotoxic potential of ifosfamide, but more importantly alerts clinicians to the potential adverse effects associated with any medication. In the case of ifosfamide-induced liver toxicity, regular monitoring of liver enzymes and other blood parameters, along with patients' clinical conditions, allows early detection of unusual side effects. The most important management strategy in patients with drug-induced liver injury is to stop treatment with the offending agent. Close monitoring and early involvement of a specialist unit are recommended, as the patient's failure to improve may necessitate intensive care input and transplantation.

## Consent

Written informed consent was obtained from the patient for publication of this case report and any accompanying images. A copy of the written consent is available for review by the Editor-in-Chief of this journal.

## Competing interests

The authors declare that they have no competing interests.

## Authors' contributions

MCMC reviewed the case notes and literature and drafted the manuscript. RLJ and IJ were responsible for the clinical care of the patient and reviewed the manuscript. IJ conceived of the article and supervised the report writing. All authors read and approved the final manuscript.
